# Scalable and Durable Brush Electrodes in Locally Enhanced Electric Field Treatment Systems for Water Disinfection

**DOI:** 10.1021/acsestengg.5c00712

**Published:** 2025-11-04

**Authors:** Feiyang Mo, Wei Wang, Shuai Wang, Nian Liu, Xing Xie

**Affiliations:** † School of Civil and Environmental Engineering, 1372 Georgia Institute of Technology, 311 Ferst Drive, Atlanta, Georgia 30332, United States; ‡ School of Chemical and Biomolecular Engineering, 1372 Georgia Institute of Technology, 311 Ferst Drive, Atlanta, Georgia 30332, United States; § Institute for Matter and Systems, 1372 Georgia Institute of Technology 345 Ferst Drive, Atlanta, Georgia 30332, United States

**Keywords:** electroporation, pulsed electric field, reactive oxygen species, stainless-steel brush electrode, water treatment reactor design

## Abstract

Locally enhanced electric field treatment (LEEFT) has emerged as a promising chlorine-free approach for water disinfection. However, its practical deployment has been limited by challenges in electrode durability and system scalability. Herein, we report a robust stainless-steel brush designed to enable long-term operation and scalability of LEEFT electrodes. A tubular reactor with coaxial electrodes featuring the brush as the center electrode was developed to combine both macroscale and microscale electric field enhancements. Operational parameters, including waveform, frequency, voltage, and flow rate, were systematically optimized to maximize microbial inactivation while minimizing metal release. Flow cytometry and control experiments revealed electroporation, assisted by reactive oxygen species, as the primary disinfection mechanism. Under optimal unipolar pulse conditions with high duty cycle and frequency, the system achieved efficient inactivation at voltages in the tens of volts range. Notably, the LEEFT system with the brush electrode has remained effective for about half a year with minimal metal release, representing a 10-fold increase in lifespan compared to previous LEEFT configurations. This work demonstrates a scalable, durable, and chemical-free solution for decentralized and sustainable water disinfection.

## Graphical abstract

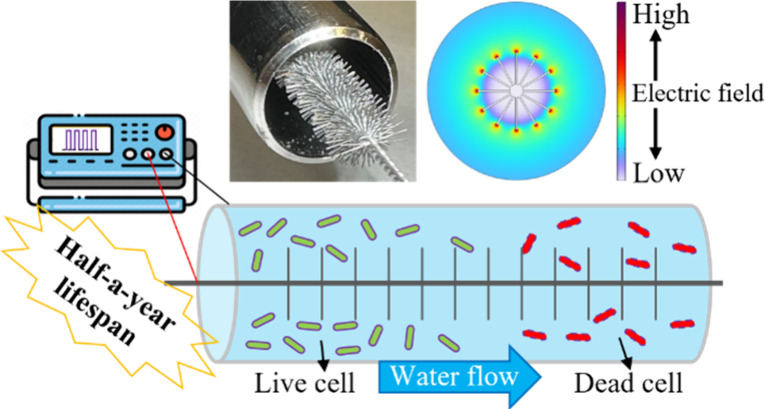

## 1 Introduction

The application of electric field treatment (EFT) for microbial inactivation dates back over a century.^1^ In the early stages, the bactericidal effects of EFT were primarily attributed to the thermal effects.^2^ The discovery of electroporation in the midtwentieth century marked a significant advancement in EFT, uncovering the nonthermal effects of electric fields on cell membranes.^3^ Specifically, when microorganisms are exposed to an external electric field, charged ions inside and outside the cell induce a transmembrane potential, which causes lipid reorientation and the formation of pores in the membrane.^4^ If the electric field strength exceeds a critical threshold (approximately 10 kV/cm), these pores become irreversible, ultimately causing microbial inactivation.^5^ To achieve high-intensity electric fields while minimizing thermal effects, EFT with high-voltage pulses within microseconds has emerged as the predominant EFT method.^6,7^

EFT has been applied in liquid food processing, such as milk, juice, and alcoholic beverages.^8–10^ In conventional EFT systems, liquid flows through a treatment chamber equipped with two electrodes spaced several millimeters apart.^11^ High-voltage electric pulses, often reaching several kilovolts, are applied across the electrodes to create a strong electric field and thus cause irreversible electroporation.^12^ EFT operates as a primarily physical process, which brings several advantages, including no disinfection byproducts (DBPs), broad spectrum, and rapid inactivation.^3,4,13,14^ However, the high voltage and short electrode spacing pose challenges, including high energy consumption and potential operational risks. These limitations have impeded the broader adoption of EFT in water disinfection, which is generally less profitable than liquid food processing.^15^

To address the challenges of conventional EFT, locally enhanced electric field treatment (LEEFT) has been developed over the past decade, demonstrating high-efficiency inactivation for water disinfection.^16^ LEEFT generates a nonuniform electric field that is low overall but significantly intensified near the electrode, thereby reducing the required voltage for microbial inactivation. This local enhancement can be achieved through two primary methods.^15^ At the macroscale, stronger electric fields can be achieved near the electrode through specific configurations (*e.g.*, coaxial electrodes).^17^ This macroscale enhancement alone is typically insufficient to cause irreversible electroporation but can increase cell membrane permeability, making bacteria more vulnerable to disinfectants like copper.^17–19^ At the microscale, electrodes modified with high-aspect-ratio one-dimensional structures (*e.g.*, nanowires) can magnify the electric field at sharp tips.^20–22^ The microscale enhancement can be several orders of magnitude and reduce the voltage to below 50 V, which is highly energy efficient.^22–26^ Nevertheless, the nanowires for microscale LEEFT remain the bottleneck, as they are prone to degradation and detachment from the substrate. To our knowledge, most of the nanowire-modified electrodes for LEEFT have a lifespan of less than 1 week.^27^ Furthermore, the complex synthesis of nanowires complicates scaling up and mass production of the LEEFT electrodes.

In this work, we introduce a rationally engineered food-grade stainless-steel brush electrode that delivers exceptional mechanical and electrochemical stability within the LEEFT system. The brush’s modular design is fully customizable and readily scalable for commercial manufacturing, obviating the need for intricate nanowire synthesis. Under optimized unipolar pulse conditions, our LEEFT system achieved sustained approximately 4-log microbial inactivation for about half a year with negligible metal leaching, a lifespan improvement of more than 10-fold compared to previous systems with nanowire-based electrodes. This robust, scalable brush electrode represents a critical step toward the practical, large-scale deployment of LEEFT water-disinfection technology.

## 2 Materials and Methods

### 2.1 Construction of Tubular Reactor with Coaxial Electrodes

We rationally designed a stainless-steel brush and had an external manufacturer (Solo Horton Brush, Inc.) fabricate it (Figure 1a). The stainless-steel brush was selected because it was the best option we could find available. Other brushes (*e.g.*, carbon brushes) in the market usually have too many bristles, which dilute the enhancement effect. The brush consisted of a central stem, 710 μm in diameter, and a plurality of bristles measuring 1.55 mm in length and 76 μm in diameter (Figure 1b). The disinfection device for LEEFT was a tubular reactor with coaxial electrodes, where a stainless-steel straw (Amazon) served as the outer electrode, and the brush was suspended inside the straw as the center electrode (Figure 1c). The reactor geometry in this study was based on previous designs with slight modifications.^17^ Both the center electrode and outer electrodes were made from 304 food-grade stainless steel, which contains approximately 18% chromium and 8% nickel. The inner diameter and length of the straw were 8.4 mm and 21.5 cm, respectively, resulting in an effective volume of approximately 10 mL (with brush volume subtracted). The reactor was flanked by two acrylic modules with plugs on both sides, ensuring that the brush electrode was securely passed through the plugs to prevent short circuits (Figure S1).

**Figure 1:**
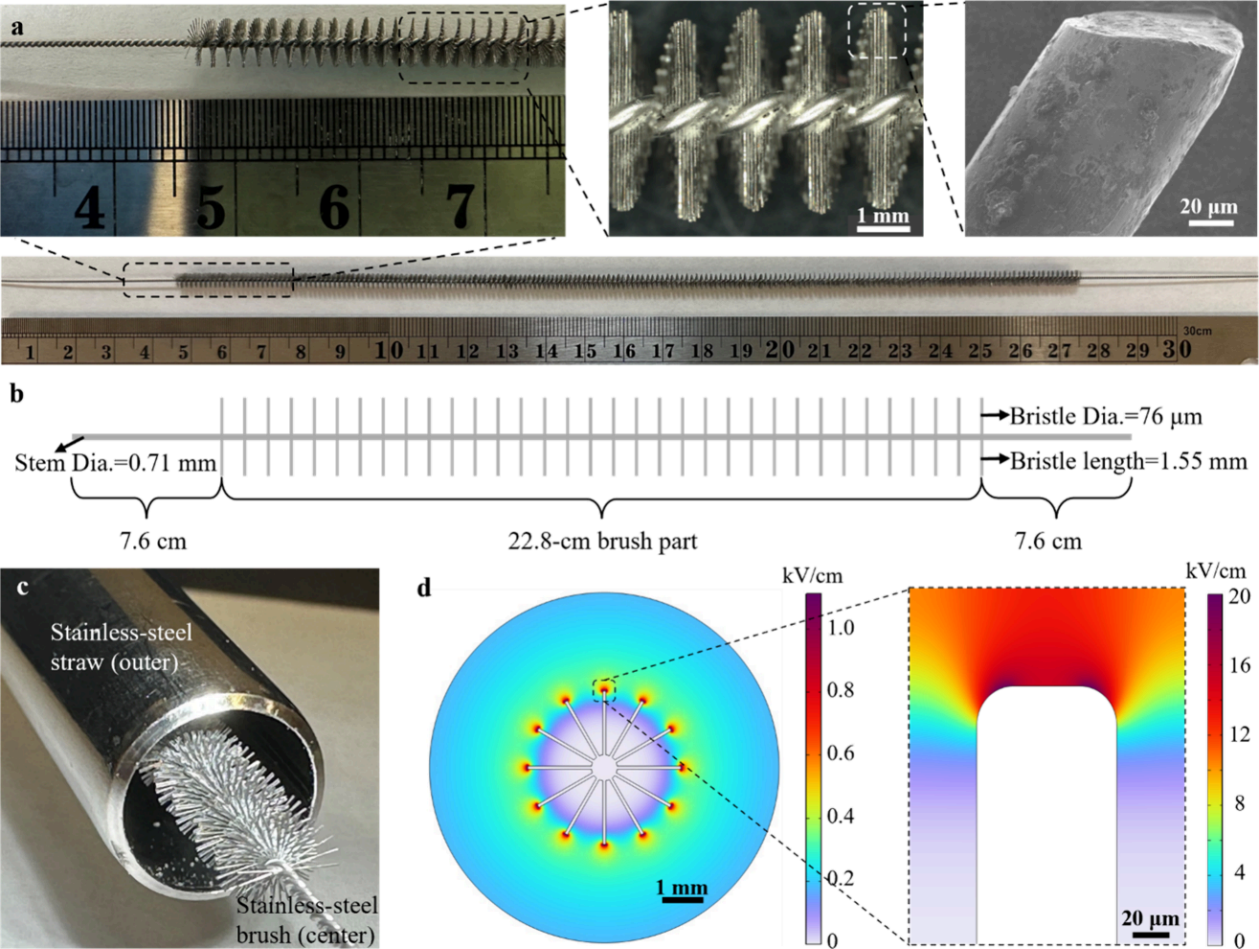
Brush electrode combines macroscale and microscale LEEFT. (a) Images of the brush electrode at different scales. (b) Schematic of the brush electrode with specifications. (c) Image of the tubular reactor with coaxial electrodes. (d) COMSOL simulation of the electric field distribution within the tubular reactor.

### 2.2 Pulse Generation

All waveforms were generated using a function generator (Keysight 33522B), which has a maximum peak-to-peak voltage of 10 V. A high-speed bipolar amplifier (NF Corporation, HSA 42014) was employed to amplify the signals to tens of volts. In this study, unipolar pulses with 25 and 50% duty cycles, bipolar pulses with 50% duty cycle, and sine waves were investigated. Unipolar pulses refer to pulses that maintain a single polarity relative to the electrode (always positive in this study), while bipolar pulses alternate between positive and negative polarities within each cycle. The duty cycle is defined as the ratio of positive pulse duration to the total period (Figure S2). The amplitude and frequency were controlled at 70 V and 500 kHz unless otherwise stated. Due to the limited bandwidth of the amplifier, signals above 500 kHz become distorted. Therefore, frequencies higher than 500 kHz were not tested in this study. An oscilloscope (Tektronix DPO 3032) equipped with a passive probe (Keysight N2843A) was used to monitor the actual voltages between the coaxial electrodes (Figure S3).

### 2.3 Bacteria Culture and Inactivation Experiments

*E. coli* (ATCC 10798) was chosen as the model bacterium for this study. Luria–Bertani (LB) broth (Miller, cat#97064) and LB agar (Miller, cat#89405) were used for the growth and culture of bacteria. *E. coli* was cultured aerobically in LB broth at 35 °C for 14–16 h to a stationary growth phase, resulting in a bacterial concentration of approximately 10^9^ CFU/mL. The bacterial solution was centrifuged at 4000 rpm for 5 min, followed by washing with deionized water three times to eliminate the effect of the broth medium. The bacterial solution was then diluted 100-fold using deionized water, serving as the influent for inactivation experiments. The influent bacterial concentration in this study (∼10^7^ CFU/mL) was selected based on previous studies with slight modifications.^17^ This concentration is intentionally much higher than that in raw water (10^2^–10^4^ CFU/mL). The electrical conductivity of the bacterial solution ranged from 0.5 to 1.0 μS/cm.

The setup for inactivation experiments is shown in Figure S3. The as-prepared bacterial solution passed through the tubular reactor, and the effluent was collected for later analysis. For the long-term test, a bacterial solution was prepared once a week for the inactivation experiment, while deionized water was used as the influent for the rest of the time. In this study, the flow rate was controlled at 2 mL/min by a peristaltic pump (MasterFlex L/S) unless otherwise stated. The standard plate count (SPC) method was used to quantify the bacterial concentration and calculate inactivation efficiency. Briefly, serially diluted samples were spread onto agar plates, which were incubated overnight at 35 °C to allow visible colony formation. Colonies were then enumerated, with each colony assumed to originate from a single viable cell, and results reported as colony-forming units (CFU) per unit volume. All SPC analyses were conducted in triplicate to ensure reliability of the results.

### 2.4 Metal Release Measurement

The concentrations of stainless-steel components, iron, chromium, and nickel, in the effluent were measured using inductively coupled plasma mass spectrometry (ICP-MS, Thermo ICAP). Calibration solutions ranging from 0 to 100 μg/L were prepared using a standard calibration solution (VWR, BDH 89800-582). Nitric acid (Sigma-Aldrich, 225711) was added to all samples at a 5% concentration to ensure complete metal dissolution.

### 2.5 Other Methods

COMSOL simulation, flow cytometry analysis, scanning electron microscope (SEM), response surface method (RSM) and electrochemical impedance spectroscopy (EIS) are detailed in Supplementary Methods S1–S5 and Tables S1 and S2.

## 3 Results and Discussion

### 3.1 Combination of Macroscale and Microscale LEEFT

The electric field distribution within the tubular reactor was modeled using COMSOL Multiphysics. As shown in Figure 1d, the LEEFT system with the brush electrode exhibited dual-level enhancement. At the macroscale, the electric field peaked near the center electrode and gradually decreased radially toward the outer electrode. At the microscale, the bristles induced the “lightning rod effect”, further enhancing the electric field at the bristle tips. As a result, the brush electrode combined macroscale and microscale LEEFT, resulting in exponentially intensified electric fields at the bristle tips (Figure 1d), which is conducive to electroporation. It is important to note that in finite element modeling, the peak field strength is highly mesh dependent, that is, when the curvature is under-resolved, the maximum is artificially suppressed. However, resolving the entire centimeter-scale reactor down to submicron or nanometer mesh size would be computationally prohibitive, leading to billions of elements. To solve this problem, a separate high-resolution model resolves only a single bristle and its immediate surroundings, which resulted in different color bars between the whole reactor and zoomed-in area (Figure 1d). For the zoomed-in regions, the largest and smallest mesh sizes were 5 × 10^–5^ and 5 × 10^–6^ cm, respectively, whereas for the whole reactor, they were 0.85 and 1.7 × 10^–6^ cm. In addition, COMSOL simulation in this study was conducted two-dimensionally to demonstrate local enhancement effects. In practice, the three-dimensional electric fields are expected to be higher than those shown in Figure 1d.

### 3.2 Selection of Waveforms and Frequency

Conventional EFT techniques typically employ high-voltage pulses with microsecond durations. However, previous LEEFT studies have not fully explored the effects of pulse polarity, duty cycle and frequency. Moreover, it remains unclear whether a sinewave, the most common form of alternating current in household applications, is effective for LEEFT. In this study, four waveforms, unipolar pulses with 25 and 50% duty cycles, bipolar pulses with 50% duty cycle, and sine waves, were applied to evaluate their impact on inactivation (Figure S2). All waveforms were set at a frequency of 500 kHz. As shown in Figure 2a, neither bipolar pulses nor sine waves were effective in inactivation even at 70 V. This inefficacy was probably attributed to two reasons. First, the rapid switching of voltage direction in bipolar waveforms prevented charge accumulation on the electrode surface, eliminating electrochemical reactions.^28^ In contrast, unipolar pulses continuously charged the electrode, allowing reactive oxygen species (ROS) generation and thus promoting bacterial inactivation.^29^ Second, bacterial transport was impaired under bipolar waveforms. The negatively charged bacteria were attracted to the center electrode when positive pulses were applied, driven by electrophoretic and dielectrophoretic forces (Video S1).^17^ Under bipolar waveforms, however, the alternating voltage caused bacteria to oscillate back and forth, preventing them from reaching the bristle tips, where the electric fields were strongest (Video S2). Only bacteria initially near the center electrode were inactivated, while the majority remained unaffected in regions with weaker electric fields. Therefore, unipolar pulses were chosen for the rest of the study.

**Figure 2:**
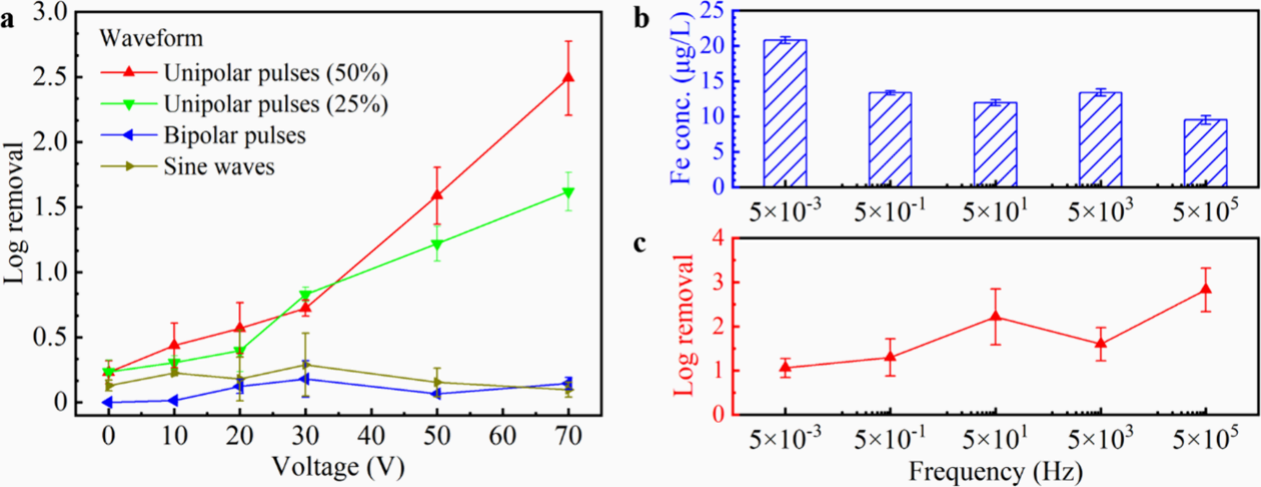
Effects of waveform and frequency on disinfection performance. (a) Inactivation efficiencies of LEEFT operated at different voltages with different waveforms and duty cycles. (b, c) Metal releases (b) and inactivation efficiencies (c) of LEEFT operated at 70 V unipolar pulses with different frequencies.

For unipolar pulses, inactivation efficiency increased with higher voltage and duty cycle (Figure 2a). This result was expected because higher voltages generated stronger electric fields while a higher duty cycle provided longer effective treatment time (hydraulic retention time still the same), both facilitating electroporation. Additionally, at a fixed pulse width of 1 μs, a 25% duty cycle results in double the rest time between pulses compared to a 50% duty cycle, allowing electrons on the electrode to discharge and thereby reducing ROS generation.^28^ The role of ROS will be discussed in the later section.^28^ It is worth noting that this study suspended bacterial samples in deionized water as a proof-of-concept demonstration for the brush electrode. In practical applications, however, the drinking water conductivity is often higher, which increases the current flowing through the system. Higher current leads to more intense electrochemical reactions and potentially causes undesirable effects like electrode corrosion, water splitting, or even ohmic heating.^28^ In such cases, employing lower-duty-cycle pulses is probably more suitable for LEEFT, which minimizes these side reactions. Future work will focus on optimizing the unipolar pulse parameters to broaden the applications of this technology, particularly in water with higher conductivity.

After optimizing the waveform and duty cycle, unipolar pulses at five different frequencies, ranging from 500 kHz to 5 mHz, were applied at 70 V to evaluate their impact on disinfection performance. All pulses maintained a duty cycle of 50%. As shown in Figure 2b, iron release in the effluent remained below 25 μg/L and decreased with increasing frequency, while chromium and nickel releases remained below 4 and 1 μg/L, respectively (Figure S4). Such low metal releases were probably attributed to the excellent electrochemical stability of stainless steel, which results from the native passive layer formed on its surface.^30^ This low level of metal release is considered safe for human exposure, highlighting the potential of this method for drinking water disinfection. In addition, a decline in iron release with frequency was observed. This is probably because under the same duty cycle, higher frequencies resulted in shorter pulse widths, and shorter pulse widths may not allow sufficient time for significant metal release since electrochemical reactions typically occur on microsecond time scales.^28^ In contrast to the iron release, the inactivation efficiency generally improved with higher frequencies (Figure 2 **c**). Response surface method (RSM) was conducted to investigate the significance of frequency and voltage (Supplementary Method S5 and Figure S5). Higher log removal observed at higher frequency was likely due to two reasons. First, higher frequencies deliver more pulses within the same treatment time, potentially leading to bacterial membrane fatigue and enhanced inactivation.^31^ Second, higher frequencies may allow more current to pass through bacteria cells. Since bacterial membranes behave like capacitors, as frequency increases, capacitive reactance decreases, lowering cell impedance and potentially boosting inactivation.^32,33^ Interestingly, the inactivation efficiency at 50 Hz was higher than 5 kHz, which deviated from the overall trend. This anomaly might be due to the balancing effects of inductive and capacitive reactance, as previously reported in the literature.^33^ The detailed mechanisms behind frequency’s impact on inactivation still require further investigation. Overall, unipolar pulses at a higher frequency exhibited more effectiveness for the LEEFT system.

### 3.3 Other Factors Affecting the Disinfection Performance

Since a single reactor achieved only a 2.5-log removal even under the optimal pulse conditions (Figure 2a), multiple reactors were connected in series to improve the inactivation efficiency (Figure S6). As shown in Figure 3a, inactivation efficiency increased with the addition of the reactors in series. With two reactors, the inactivation efficiency reached 3.9 logs, and with three reactors, the inactivation further increased to 4.6 logs. This result was expected since more reactors treated the influent multiple times, which promoted inactivation. However, the incremental gain in log removal decreased with more reactors in series. This reduction might be attributed to the sample remixing between reactors after each stage, which could partially impede live bacteria from reaching the center electrode for effective inactivation. It is worth mentioning that in principle, connecting reactors in series is equivalent to operating a single elongated reactor, and the latter would be more effective in enhancing inactivation efficiency because it avoids remixing. Unfortunately, the total brush length could not be increased at current stage due to manufacturing limitations. Given that two reactors have already achieved nearly 4-log removal, and an additional reactor only contributed less than 1-log improvement in activation, we connected two reactors in series for the later experiments for simplicity.

**Figure 3:**
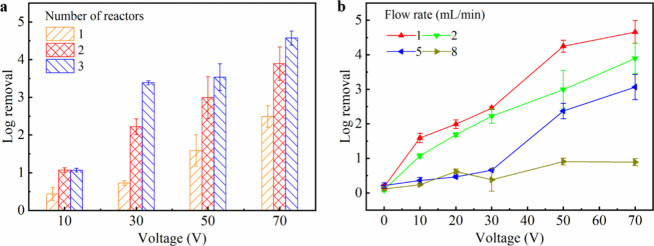
Effects of the number of reactors in series and flow rate on disinfection performance. (a) Inactivation efficiencies gained with different numbers of LEEFT reactors in series. (b) Inactivation efficiencies of LEEFT operated with different voltages and flow rates.

The effects of the flow rate on the LEEFT system were also investigated. As shown in Figure 3b, the inactivation efficiency increased with lower flow rates. This result was expected because a lower flow rate increased hydraulic retention time (HRT), facilitating electroporation. In addition, longer HRT also allowed more bacteria to be drawn toward the brush electrode, resulting in higher inactivation efficiency. For instance, at 30 V, the LEEFT system achieved over 2-log removal at the flow rate of 1 and 2 mL/min (*i.e.*, HRT of 10 and 5 min), while inactivation dropped to less than 1-log at 5 and 8 mL/min (*i.e.*, HRT of 2 and 1.25 min). Notably, at the flow rate of 8 mL/min, the inactivation efficiency remained below 1-log even at 70 V. This was probably because the bacteria were flushed out of the reactor before reaching the bristle tips, which also aligned with results of bacterial transport simulation (Video S1). It is important to clarify that the HRT here reflects bacterial transport rather than the true contact time, since electroporation itself occurs on the order of microseconds to milliseconds.^14^ For our current LEEFT device, bacteria are mainly driven by electrophoretic forces to the center brush electrode, and a retention time of a few minutes is required, limiting the treatment throughput. Future work will explore to improve the mixing conditions, such as introducing rationally designed baffles or internal flow perturbation elements to disrupt laminar streamlines, thereby reducing HRT and enabling higher flow rates.

### 3.4 Inactivation Mechanisms

Although the “lightning rod effect” is theoretically applicable across all scales, this study represents the first application of micrometer-scale structures (*i.e.*, bristles) to modify the LEEFT electrode. Control experiments with a stainless-steel rod and stem were conducted to investigate the effectiveness of the bristle modification. The diameters of the rod and stem were 3.16 and 0.71 mm, respectively, while their lengths were both 38 cm, the same as the brush (Supplementary Method 1). As shown in Figure 4a, the brush electrode achieved an inactivation efficiency 2.4-log higher than that of the rod electrode. Notably, the system impedance of the rod electrode was comparable to that of the brush electrode (Figure S7), and their macroscopic electric field distributions were also similar (Figures 1d and[Fig fig4]a, inset). Therefore, the enhanced inactivation efficiency of the brush electrode is likely attributed to the microscale LEEFT induced by the bristles, which facilitates electroporation. Interestingly, the stem electrode without bristles achieved only a 0.9-log removal despite exhibiting a higher electric field near the stem than the rod (Figure 4a, inset). This discrepancy is likely due to the increase in system impedance caused by the stem volume change (Figure S7), which may influence electrochemical reactions such as ROS generation. The remaining inactivation observed in the control experiments is likely attributed to direct oxidation or the effects of ROS.^17,34^ Overall, micrometer-scale bristles could effectively enhance inactivation efficiency. However, unlike previous nanowire-modified electrodes, bristle modification alters electrolyte volume and interelectrode distance at the macroscale, which should be considered in future electrode designs.

**Figure 4:**
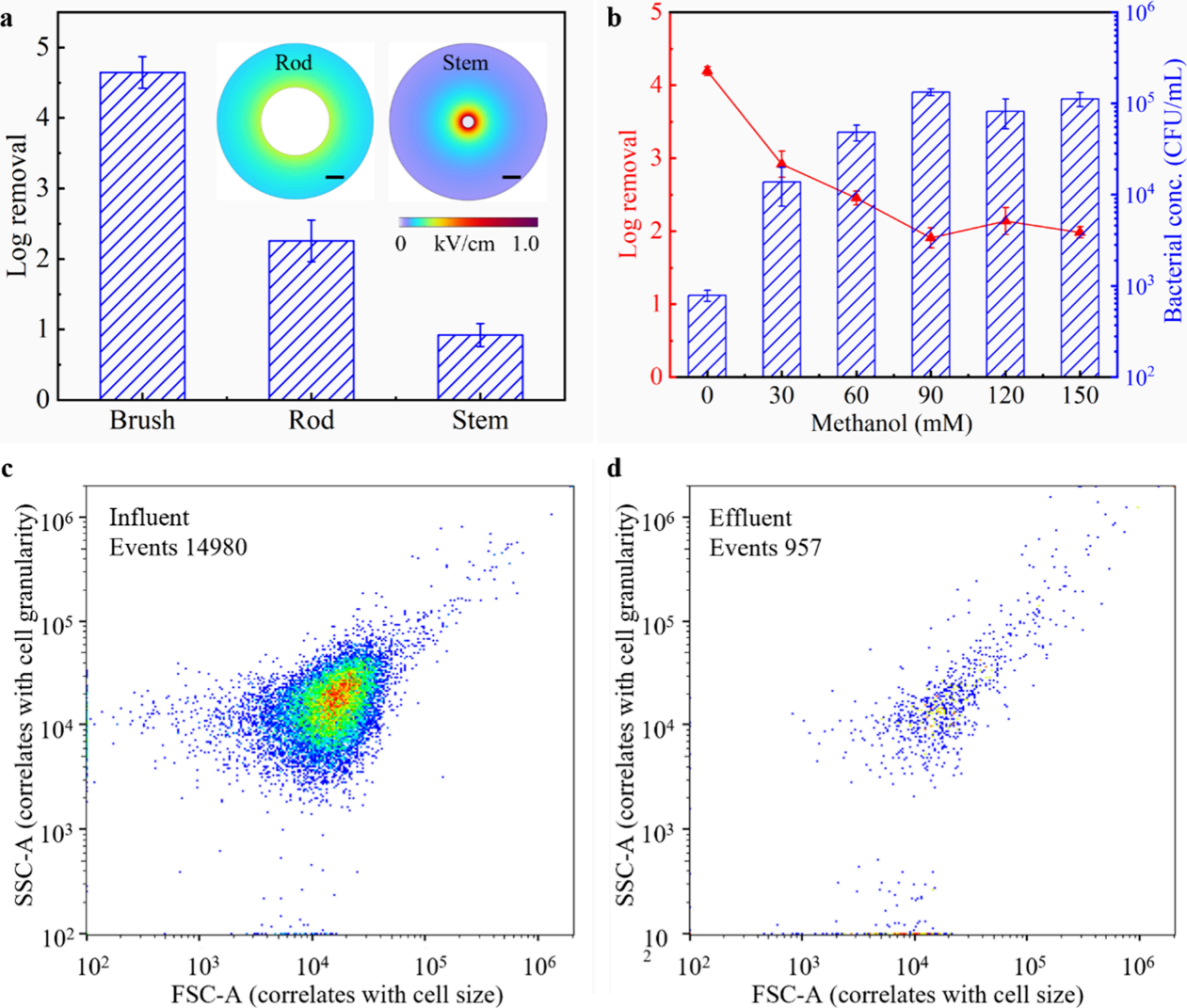
Investigation of inactivation mechanisms. (a) Inactivation efficiencies of LEEFT with different center electrodes at 70 V. Inset, COMSOL simulations of the electric field distribution within the tubular reactor with the rod (left) and stem (right) electrodes, and the scale bars represent 1 mm. (b) Effects of surface radical scavenger on inactivation efficiency. (c, d) Flow cytometry dot plots of bacteria before (c) and after (d) LEEFT.

Based on our previous studies, electroporation is hypothesized to be the primary mechanism of microbial inactivation in EFT, but other factors may also contribute to the bactericidal effect, including ohmic heating, ROS, and electrosorption.^35^ Since no significant temperature change or bacterial accumulation on the electrode was observed during the LEEFT experiments, the contributions of ohmic heating and electrosorption can be ruled out. To assess the role of ROS, methanol, a common surface radical scavenger, was introduced.^36,37^ Control experiments confirmed that methanol alone did not affect bacterial concentration (Figure S8). As shown in Figure 4b, the log removal decreased as the methanol concentration increased from 0 to 90 mM and remained stable thereafter, indicating that the ROS play a role in the inactivation mechanism. It is worth mentioning that previous LEEFT studies have demonstrated synergistic effects between electroporation and other disinfectants, such as copper, ozone, and chlorine.^38–40^ In this study, the approximately 2-log reduction in activation efficiency after the addition of methanol should be attributed to both ROS alone and the synergistic effect between ROS and electroporation. However, as ROS is transient and undetectable in the effluent, their specific function within the LEEFT system requires further investigation.^41^ Notably, even when the methanol concentration exceeded 90 mM, completely inhibiting ROS, the LEEFT system still achieved a 2-log reduction, confirming that electroporation remains a crucial mechanism for microbial inactivation. In addition, when LEEFT operates under conditions where ROS are formed, a minimal amount of DBPs may also be generated. However, the investigation of DBPs lies beyond the scope of this study.

Flow cytometry analysis and SEM were performed to assess bacterial conditions before and after LEEFT. As shown in Figure 4c,d, the bacterial events in the effluent were 1.2-log lower than those in the influent, indicating that approximately 6% of bacteria with relatively integral structures (whether alive or dead) remained in the effluent. SEM images of the remaining bacteria after LEEFT further revealed severe membrane damage (Figures S9 and S10), suggesting that cell lysis occurred within the reactor. This membrane disruption was likely attributed to ROS activity, as ROS are known to cause extensive structural damage to bacterial membranes.^41,42^ This reduction observed in flow cytometry also aligns with the methanol control experiments, which demonstrated that ROS contributed to less than 2-log bacterial removal (Figure 4b). Notably, standard plate count analysis revealed an approximately 4-log reduction (Figure 3, indicating that only 0.01% of bacteria remained viable post-LEEFT. The discrepancy between standard plate count and flow cytometry analysis is likely due to different inactivation mechanisms of ROS and electroporation. Specifically, the electroporated cells retain relatively integral membranes and can still be detected by flow cytometry, whereas ROS-induced damage leads to membrane fragmentation, rendering the cells undetectable.^40,43^ Overall, the dominant inactivation mechanism was electroporation while ROS did assist to promote the inactivation efficiency.

### 3.5 Long-Term Operation

Lastly, a long-term test was conducted to assess the reliability of the LEEFT system. As shown in Figure 5, the LEEFT system operated continuously for about half a year, maintaining a stable 4-log inactivation efficiency with slight fluctuations. The concentrations of iron, chromium, and nickel were monitored throughout the long-term operation. The results showed that iron and chromium concentrations basically remained below 10 μg/L, while nickel release was negligible (Figure S11). Given that the USEPA regulatory limit for chromium is 100 μg/L and advisory guidelines for iron and nickel are 300 and 100 μg/L, respectively, the observed metal release from the brush electrode remains well within safe levels for human exposure, making it suitable for drinking water disinfection.^44–46^ Furthermore, both inactivation efficiency and metal release during long-term operation remained consistent with those observed in the fresh-prepared device (Figures 2b and 3b), exhibiting excellent reliability of the system. Notably, in previous studies, the most stable LEEFT electrode, PDA-coated Cu_3_PNW-modified copper foam, sustained continuous operation for 15 days, while most other LEEFT electrodes failed within a week.^27,47^ In contrast, this brush electrode demonstrated exceptional durability, extending the operational lifespan exponentially. This significant improvement enhances the feasibility of LEEFT technology for long-term applications.

**Figure 5:**
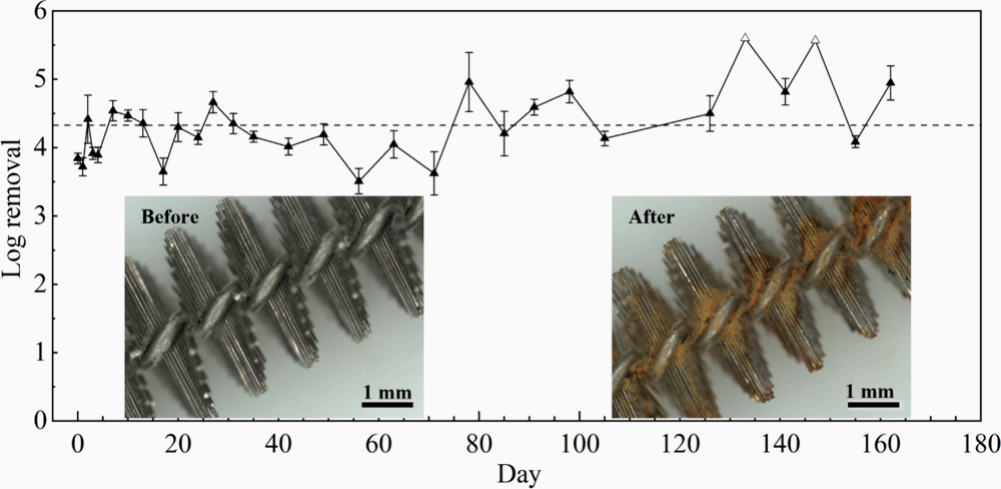
Long-term performance for the LEEFT system with the brush electrode for bacteria inactivation. The hollow points indicate values when the detection limit was reached. The dash line indicates the average value. Inset, the images of the brush electrode before (left) and after (right) LEEFT.

To further assess electrode degradation, the broken brush electrode after long-term operation was characterized using digital microscopy, SEM, and EDS analysis. As shown in Figure 5, inset, after about six-month operation, significant rusting was observed, particularly at the junction between the bristles and the stem. The brush electrode in the second reactor fractured at one end, and some loosened parts of the stem were also detected at the end of the brush electrode in the first reactor (Figure S12). The more severe corrosion at the end was probably because of the configuration of the tubular reactor with coaxial electrodes where a higher current passed through the brush electrode in the plastic module (Figure S13). EDS analysis revealed an increased proportion of oxygen after LEEFT (Figures S14 and S15), indicating the corrosion probably resulted from electrochemical oxidation. Since the breakage occurred at the end of the electrode while the main structure remained intact, future research could focus on reinforcing the electrode ends to improve durability, as well as removing rust to facilitate reuse.

### 3.6 Advantages, Limitations, and Future Work

As a novel chlorine-free technique, LEEFT has attracted considerable attention over the past decade.^27^ Despite its proven efficacy and the absence of DBP formation, LEEFT application remains confined to the laboratory due to key scalability challenges. Macroscale LEEFT typically requires additional disinfectants such as copper to achieve sufficient microbial inactivation, while microscale LEEFT faces limitations related to electrode longevity and scalability stemming from the complexity of nanowire synthesis.^17,27^ The brush electrode in this study addresses these challenges by integrating both macroscale and microscale LEEFT. This brush electrode enables effective disinfection without supplementary chemical disinfectants while offering superior mechanical and electrochemical stability. This significantly extends electrode lifespan and enhances system reliability. Additionally, the scalable and customizable brush design eliminates the need for intricate nanowire synthesis, making it suitable for mass production and commercialization.

Despite these advancements, there are still some limitations for this study. First, only *E. coli* was selected as the model organism, without the inclusion of Gram-positive bacteria. Although the efficacy of LEEFT against both Gram-negative and Gram-positive bacteria has been extensively reported in previous studies, future work will include testing additional microorganisms, such as antibiotic-resistant bacteria and algae, to further evaluate its broad applicability.^19,47^ Second, the system with the brush electrode required operating voltages in the tens of volts range for effective microbial inactivation. Such voltage is higher than previous LEEFTT studies but remains several orders of magnitude lower than conventional EFT.^22–24,35^ This increased voltage is attributed to the smaller aspect ratio of the bristles compared to previous nanowire-modified electrodes, leading to weaker microscale electric field enhancement. Future research should focus on optimizing brush design to further reduce the required operating voltage. Lastly, this study employed deionized water as the test matrix for proof-of-concept. The conductivity of the bacterial suspension in this study (0.5–1 μS/cm) is lower than that of typical drinking water (50–200 μS/cm). The low conductivity helps suppress electrochemical side reactions, allowing the electroporation, an electrophysical process, to be investigated more directly. To ensure broader practical applicability, future studies will investigate the impacts of dissolved organic matters, turbidity, ionic strength, and pH under environmentally relevant conditions to better assess the applicability of LEEFT for real water. For example, higher conductivity may enhance electrochemical side reactions and promote ohmic heating, while pH can influence both bacterial surface charge and ROS generation, thereby affecting inactivation efficiency. In addition, higher water hardness may increase electrode fouling due to the formation of precipitates, whereas elevated temperature can accelerate corrosion, ultimately reducing the lifespan of the brush electrode. Addressing these challenges will be critical for advancing LEEFT technology toward large-scale, sustainable water disinfection solutions.

## 4 Conclusions

In this study, we introduced a rationally engineered food-grade stainless-steel brush electrode that delivers exceptional mechanical and electrochemical stability within the LEEFT system. The coaxial configuration and bristles on the brush provided macroscale and microscale electric field enhancements, respectively, intensifying the electric field at bristle tips to achieve electroporation with only tens of volts, over 2 orders of magnitude lower than conventional EFT. Unipolar pulses with high duty cycle and high frequency were selected as the optimal conditions to power the LEEFT system, which exhibited superior inactivation efficiency over 4-log removal with minimal metal release. Other factors, including high voltage, low flow rate, and multiple reactors, also promoted the performance of the LLEFT system. Control tests with ROS quenchers, flow cytometry, and SEM confirmed electroporation as the dominant inactivation mechanism, supplemented by ROS. Remarkably, our LEEFT system with the brush electrode has sustained stable performance for about half a year, representing a 10-fold increase in lifespan compared to previous LEEFT studies. The electrochemical corrosion of the brush electrode was the main reason for system failure, but the main brush structure remained intact afterward. In general, this study provided a viable engineering solution for durable and scalable LEEFT electrode design. Future work will focus on evaluating system performance in real water matrices with higher conductivity and organic content.

## Supplementary Material







## Data Availability

Data will be made available on request.

## Notes

Supplementary methods 1–5 include EIS, flow cytometry, SEM, COMSOL simulations, and RSM with supplementary reference; supplementary tables 1 and 2 show the parameters of COMSOL simulations and regression coefficients of RSM, respectively; and supplementary figures 1–15 show experimental setup, electrode and bacterial characterizations, and ICP measurements (PDF)
Video S1 (MP4)
Video S2 (MP4)
