# Supplementary material for: Scalable and Durable Brush Electrodes in Locally Enhanced Electric Field Treatment Systems for Water Disinfection
Source: ACS ES T Eng. 2025 Nov 4;6(1):124–32. doi: 10.1021/acsestengg.5c00712 (PMC12797229; doi:10.1021/acsestengg.5c00712)
Supplement: Supplementary file 1 [file ee5c00712_si_001.pdf]

Supporting information for

Scalable and Durable Brush Electrodes in Locally Enhanced Electric Field Treatment Systems for Water Disinfection

Feiyang Mo,[†] Wei Wang,[†] Shuai Wang,[†] Nian Liu[*,‡] and Xing Xie[*,†,§]

[†]School of Civil and Environmental Engineering, Georgia Institute of Technology, 311 Ferst Drive, Atlanta, Georgia 30332, United States

[‡]School of Chemical and Biomolecular Engineering, Georgia Institute of Technology, 311 Ferst Drive, Atlanta, Georgia 30332, United States

[§]Institute for Matter and Systems, Georgia Institute of Technology, 345 Ferst Drive, Atlanta, Georgia 30332, United States

*Email: nian.liu@chbe.gatech.edu

*Email: xing.xie@ce.gatech.edu

This file includes:

Supplementary Methods 1 – 5

Supplementary Tables S1 - 2

Supplementary Figures S1 – S15

Supplementary Reference

Supplementary Methods

1. Control Experiments and Electrochemical Impedance Spectroscopy (EIS)

A stainless-steel rod and stem with a diameter of 3.16 mm and 0.71 mm, respectively, were used as the control electrode for the brush. The length of the rod and stem was 38 cm, the same as the brush. All the setup for control experiments is the same as that for the brush electrode. Unipolar pulses with 50% duty cycle and 500 kHz were applied to power the system. The voltage and flow rate for the control experiments were 70 V and 2 mL/min, respectively.

For EIS, the tubular reactor was connected to a potentiostat (BioLogic VMP3). EIS was conducted under a two-electrode system to measure the impedance of the whole LEEFT system. The electrolyte in the reactor was Milli-Q water, whose conductivity was close to the bacterial solution used in the LEEFT experiments. The current was set at 100 μA, while the frequency ranged from 1 MHz to 100 mHz.

2. Flow Cytometry Analysis

One milliliter of influent and effluent samples was collected and stained with 10 μM SYTO 9 (Invitrogen) and 30 μM propidium iodide (PI, Invitrogen).[1] SYTO 9 can permeate both live and dead cells, while PI only enters cells with compromised membranes. After staining, the samples were incubated in the dark at room temperature for 10 min and analyzed using a CytoFlex S (Beckman Coulter). The sample was taken within a fixed time, so the volume was the same for each sample. An FSC-A threshold of 5000 was set to eliminate the effects of background noise. The results were processed using FlowJo software.

3. Bacterial Sample Preparation for Scanning Electron Microscope (SEM)

All bacterial samples for SEM were harvested by centrifugation at 4000 rpm for 5 min, and the supernatant was then removed. The bacteria were fixed in a 0.1 M phosphate-buffered solution (pH 7.3) containing 2% glutaraldehyde.[2] The samples were then dehydrated in a series of ethanol solutions with increasing concentrations (50%, 75%, and 100%), followed by drying in 100% ethanol.[2] Finally, the samples were dispersed onto copper tape and coated with Au (Hummer 6 Sputterer) for SEM characterization (Hitachi SU8230).

4. Electric Field Simulation

The simulation of charged bacteria motion in coupled electric and fluid fields was conducted using COMSOL Multiphysics.[3] The parameters used for the simulation are shown in (**Table S1**). The device configuration was reconstructed using the parameters of the real tubular reactor. The Electrostatics modules were used to simulate the electric field distribution within the device. Enhanced fields near the center electrode were captured through local mesh refinement. The pulse was generated by the wave function.

The Particle Tracing for Fluid Flow and Electrostatics modules were employed to model the motion of bacteria under the influence of electric fields and fluid dynamics. Due to computational limitations, the pulse width was simplified to 1 ms with rise and fall times of 0.1 ms. The fluid was treated as an incompressible Newtonian medium with laminar flow, and the electric field was generated by setting appropriate boundary conditions on the electrodes. Bacteria were introduced into the flow field with predefined size, charge, and density distributions. The governing equations incorporated drag force, electrostatic force, and Brownian motion, reflecting particle behavior

under various field conditions. Bacteria trajectories and velocities were evaluated over time, and a time-dependent solver was used to ensure accurate tracking of transient behaviors. The model was validated by comparing simulated results with experimental observations.

Table S1. The parameters used for COMSOL simulation

Parameter	Unit	Value
Pulse duty cycle	%	50
Pulse period	s	10^{-3}
Fall and rise time	s	10^{-4}
Pulse amplitude	V	70
Electrical conductivity of medium	S/m	5.5×10^{-6}
Relative permittivity of medium	1	80
Electrical conductivity of electrode	S/m	1.4×10^{6}
Relative permittivity of electrode	1	1
Mass of a particle	g	10^{-12}
Diameter of a particle	m	2×10^{-6}
Vacuum permittivity	F/m	8.85×10^{-12}
Relative permittivity of particle	1	60
Charge number of bacteria	1	-100
Inlet particle number	1	100
Length of the outer electrode	cm	21.5
Diameter of the outer electrode	cm	0.84
Diameter of the stem	µm	710
Length of the bristle	µm	1550
Diameter of the bristle	µm	76

5. Response Surface Method (RSM)

RSM was applied to assess the significance of frequency and voltage using MATLAB R2024a. Given that the dataset was limited, a simplified model was adopted that included only main effects and their interactions, without quadratic terms. To address the large difference in magnitude between variables, frequency was transformed to a logarithmic scale. The resulting model can be expressed as follows.

$$\text{Log removal} = X_0 + X_1 \cdot \text{Voltage} + X_2 \cdot \text{LogFreq} + X_3 \cdot \text{Voltage} \cdot \text{LogFreq}$$

The regression coefficients were summarized in **Table S2**, and the fitted response surface was illustrated in **Figure S5**. The corresponding MATLAB code was attached below.

```
% Data
Voltage   = [70;70;70;70;70;0;10;20;30;50;70];
Frequency = [5e5;5e3;5e1;5e-1;5e-3;5e5;5e5;5e5;5e5;5e5;5e5];
LogRemoval= [2.83;1.60;2.22;1.30;1.06;0.23;0.44;0.57;0.72;1.59;2.49];
% Transform frequency to log-scale
logFreq = log10(Frequency);
% Create table for regression
tbl = table(Voltage, logFreq, LogRemoval);
% Fit response surface model (linear, interaction, no quadratic terms)
mdl = fitlm(tbl, 'LogRemoval ~ Voltage*logFreq + Voltage + logFreq');
disp(mdl);
```

As shown in **Table S2**, voltage exhibited a highly significant effect on log removal efficiency ($p < 0.001$), confirming that inactivation is strongly influenced by voltage. In contrast, frequency alone was not significant ($p \approx 0.98$). However, the interaction between voltage and frequency was relatively significant ($p \approx 0.021$), suggesting that the impact of voltage depends on the operating

frequency. This trend is consistent with the observations in **Figure 2c**. Moreover, the intercept was estimated as zero, which is reasonable given that negligible inactivation was observed at zero voltage. The model demonstrated a good fit, with a relatively small residual and an R^2 of approximately 0.89, indicating that it explains most of the observed variation.

Table S2. The regression coefficient

Coefficient	Term	Estimate	SE	tState	pValue
X_0	Intercept	0	0	NaN	NaN
X_1	Voltage	2.10×10^{-2}	2.41×10^{-3}	8.71	5.26×10^{-5}
X_2	LogFreq	-7.81×10^{-4}	3.61×10^{-2}	-2.17×10^{-2}	0.98
X_3	Voltage×LogFreq	2.44×10^{-3}	8.60×10^{-4}	2.84	0.025

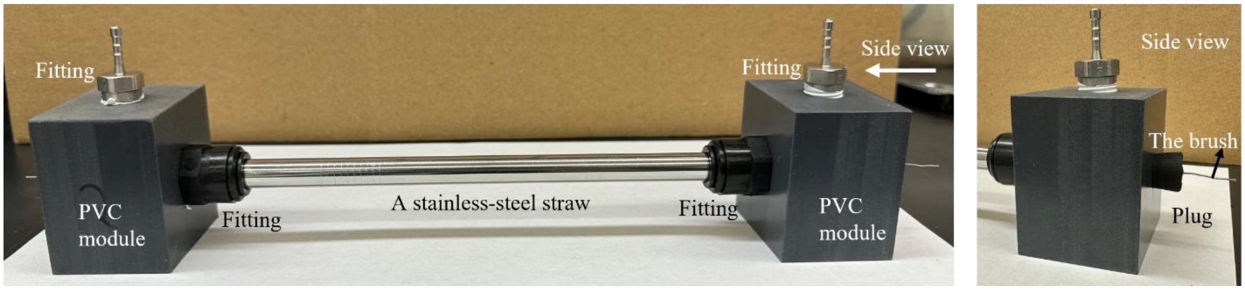

Figure S1. The tubular reactor as the LEEFT disinfection device.

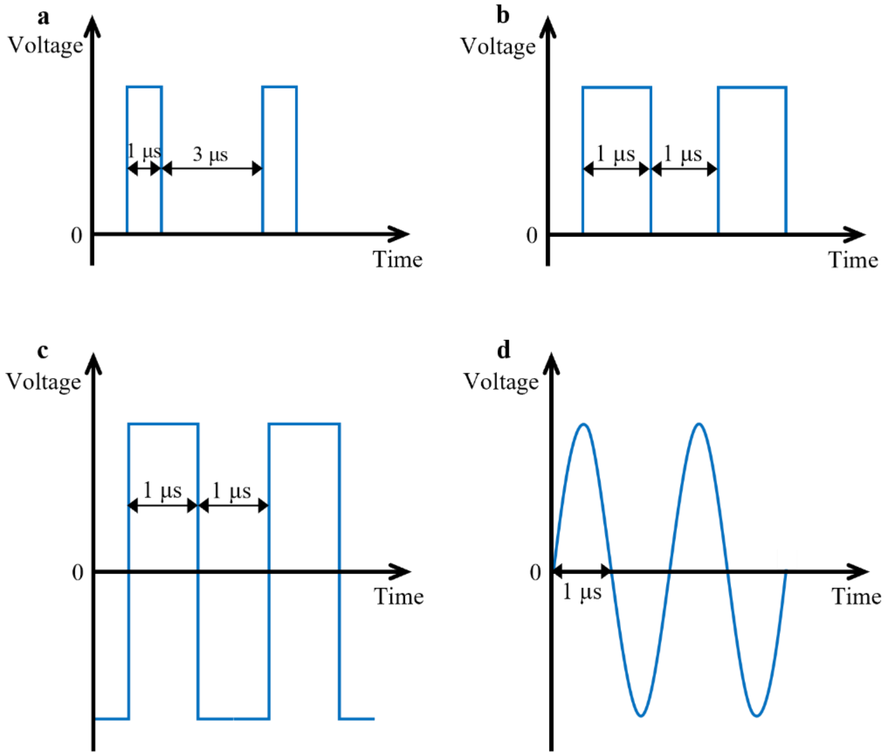

Figure S2. Four different waveforms at the frequency of 500 kHz. (a) Unipolar pulses with 25% duty cycle. (b) Unipolar pulses with 50% duty cycle. (c) Bipolar pulses with 50% duty cycle. (d) Sine waves.

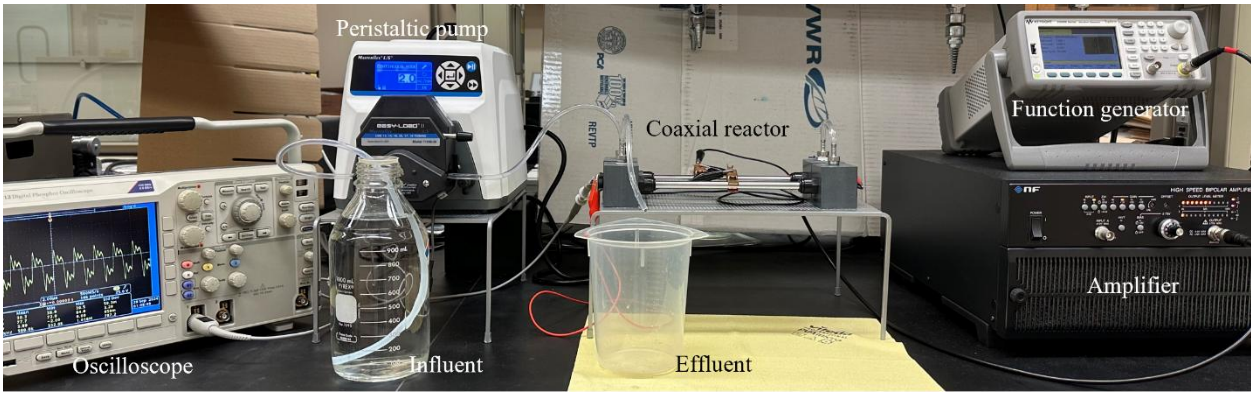

Figure S3. The experimental setup of the LEEFT.

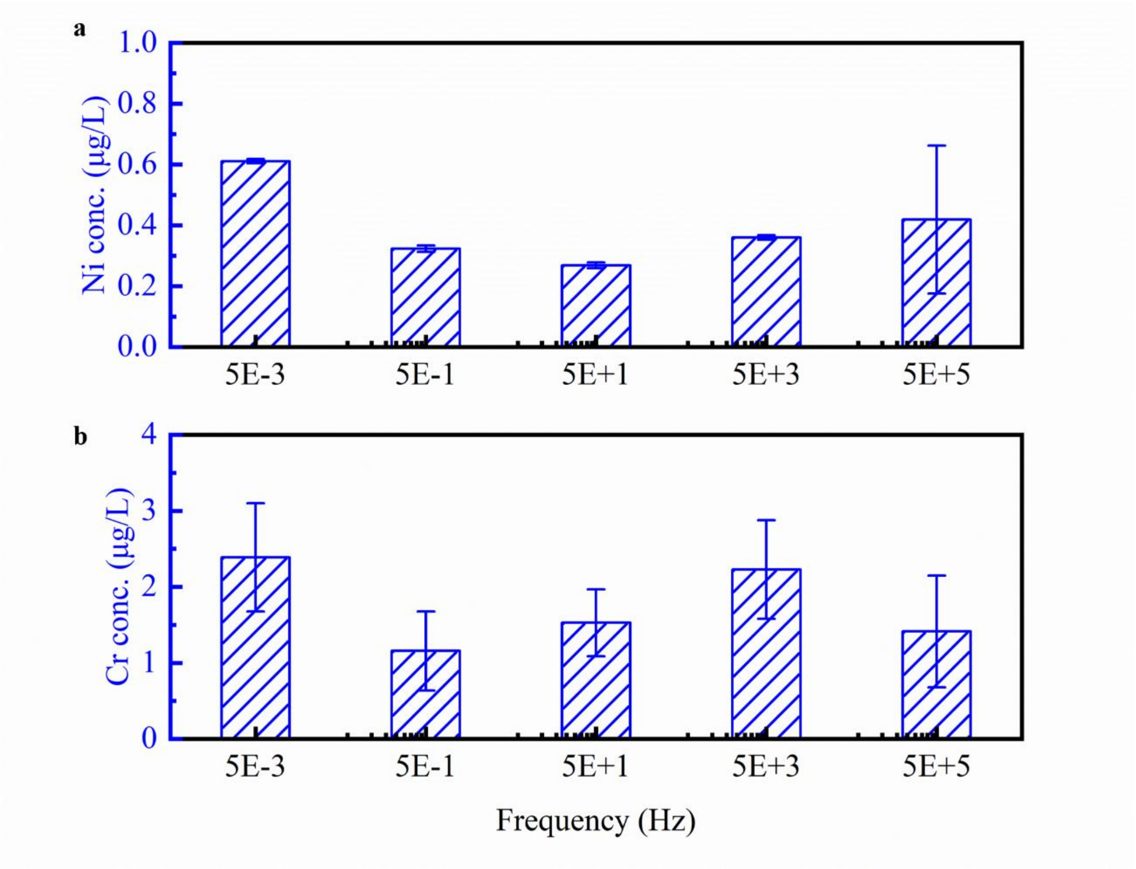

Figure S4. (a) Nickel and (b) chromium releases of LEEFT operated at 70 V unipolar pulses with different frequencies.

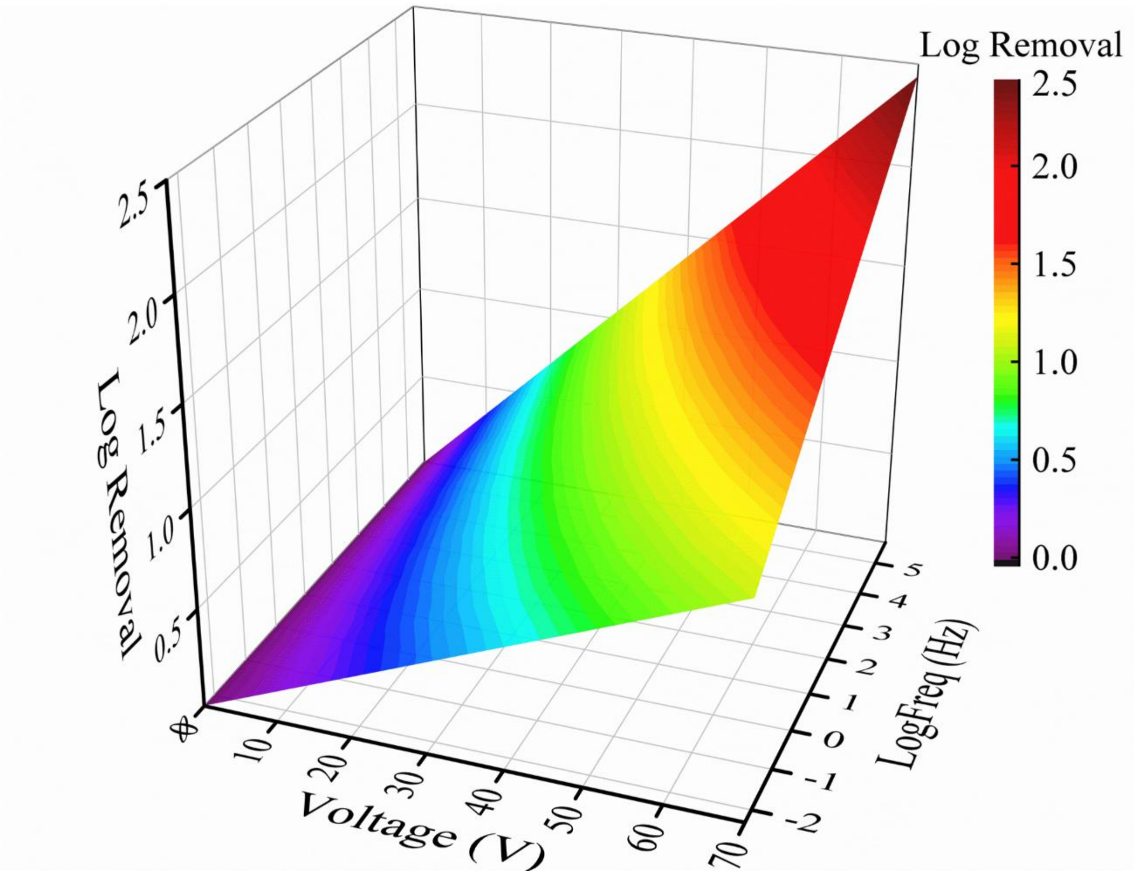

119

120 **Figure S5.** The response surface plot showing the effects of frequency and voltage on inactivation

121 efficiency.

122

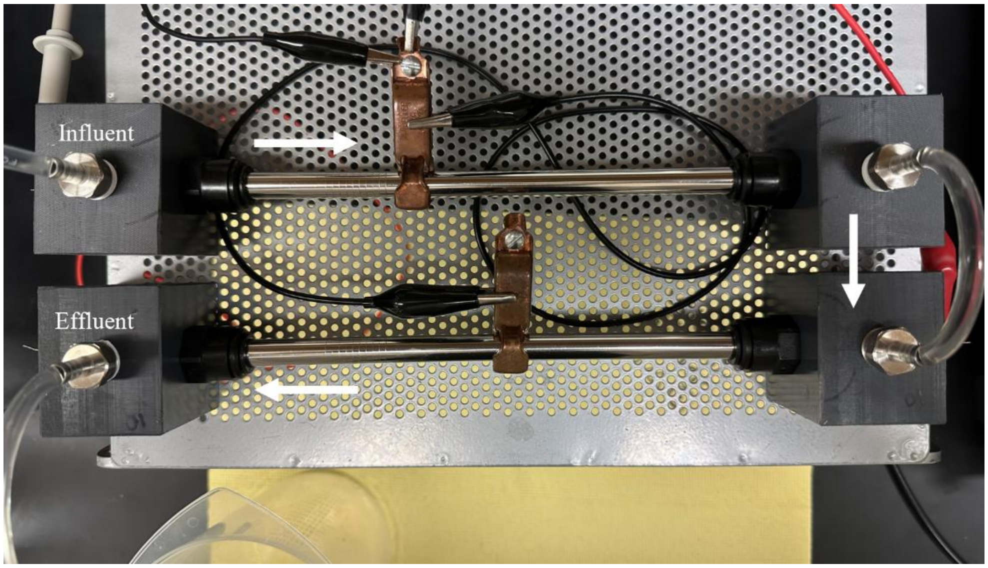

Figure S6. Two tubular reactors connected in series.

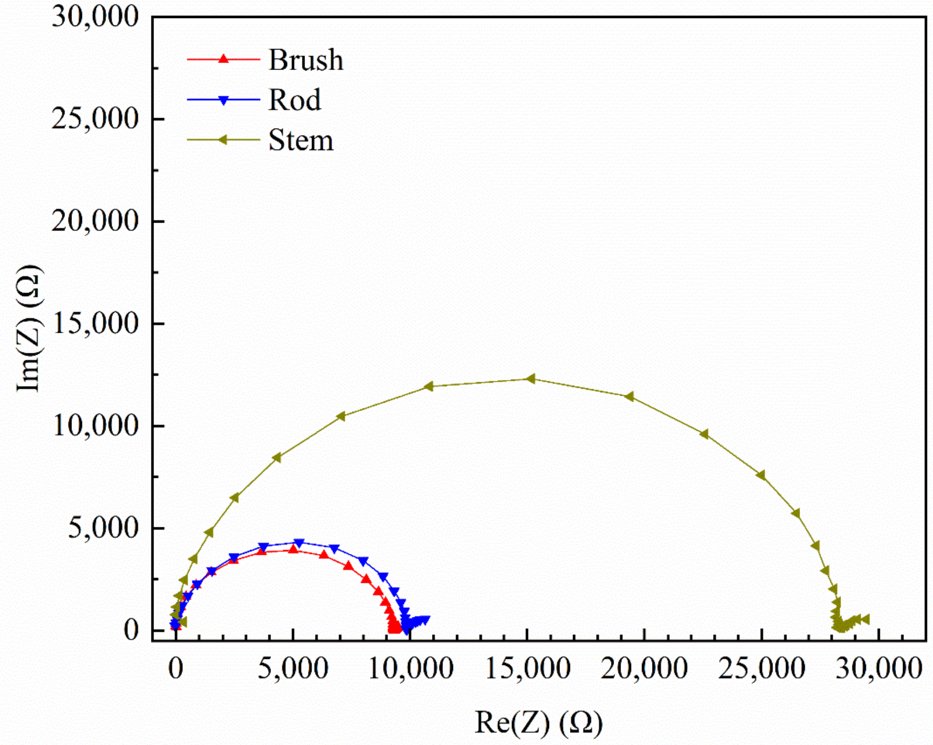

Figure S7. Nyquist plot of the LEEFT system with different center electrodes.

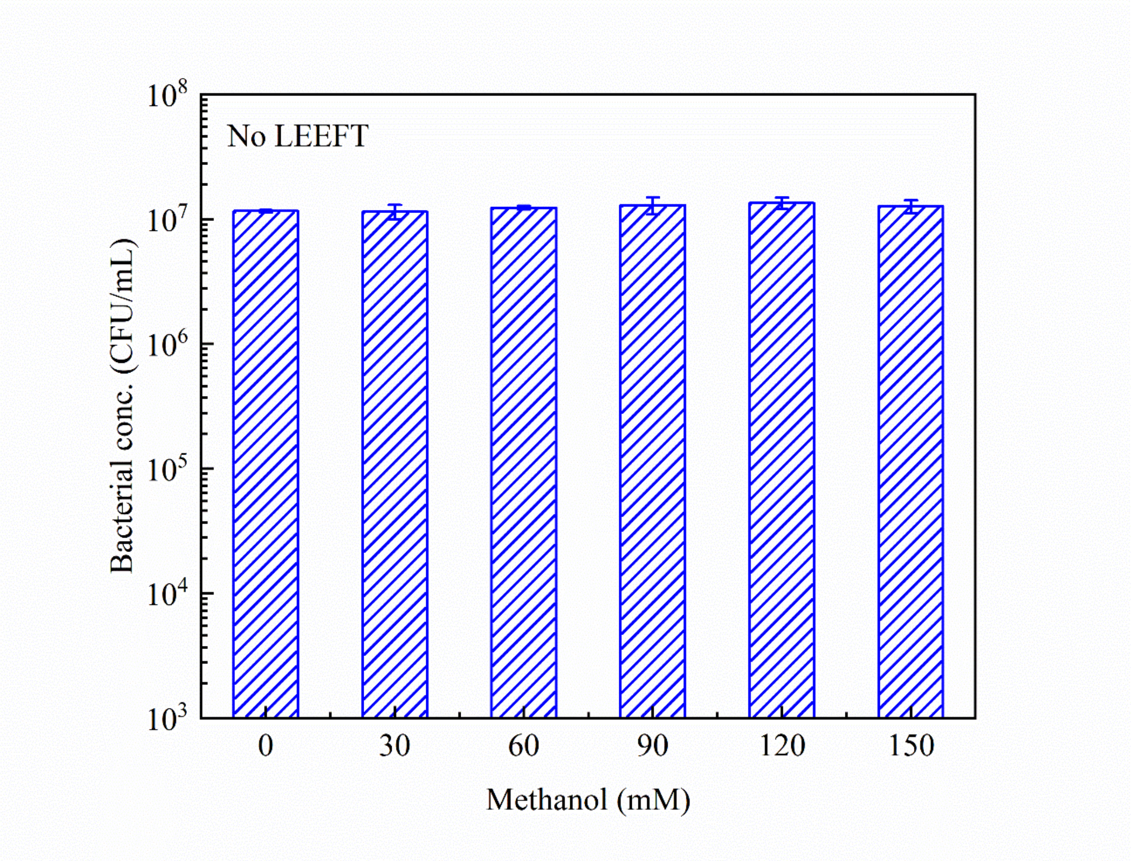

Figure S8. Bacterial concentration after different concentrations of methanol addition.

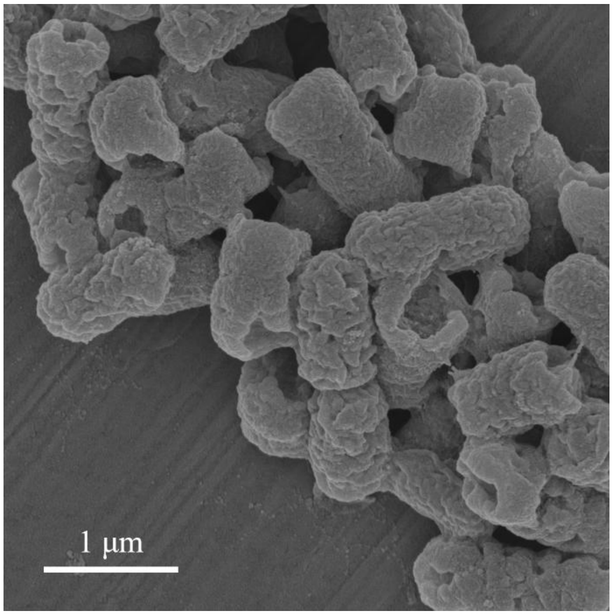

Figure S9. SEM image of the bacteria in the influent. The raw bacteria show some pores and incomplete structures, probably because the bacteria in this study are in the stationary phase.

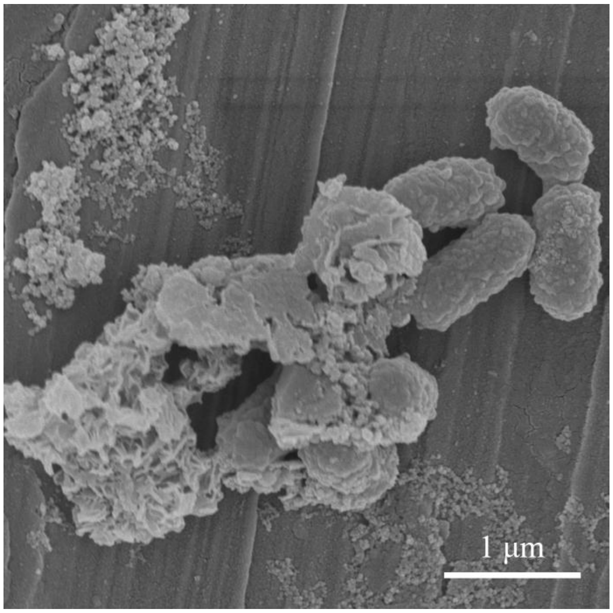

Figure S10. SEM image of the bacteria in the effluent.

140 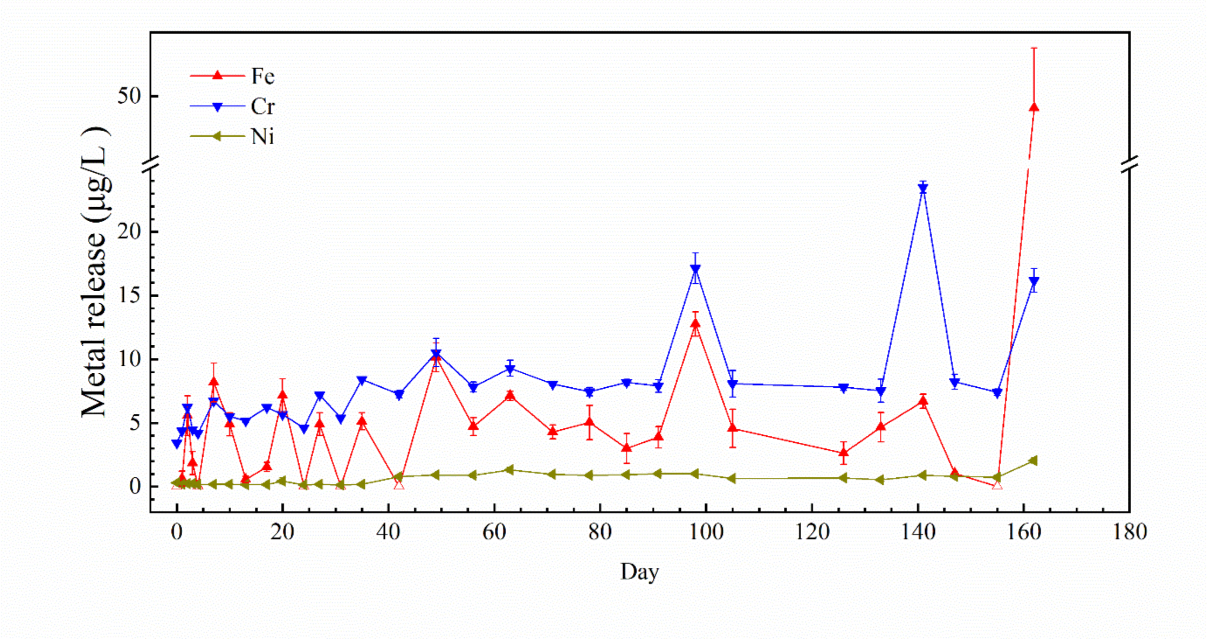

141 **Figure S11.** Metal release during the long-term operation.

142

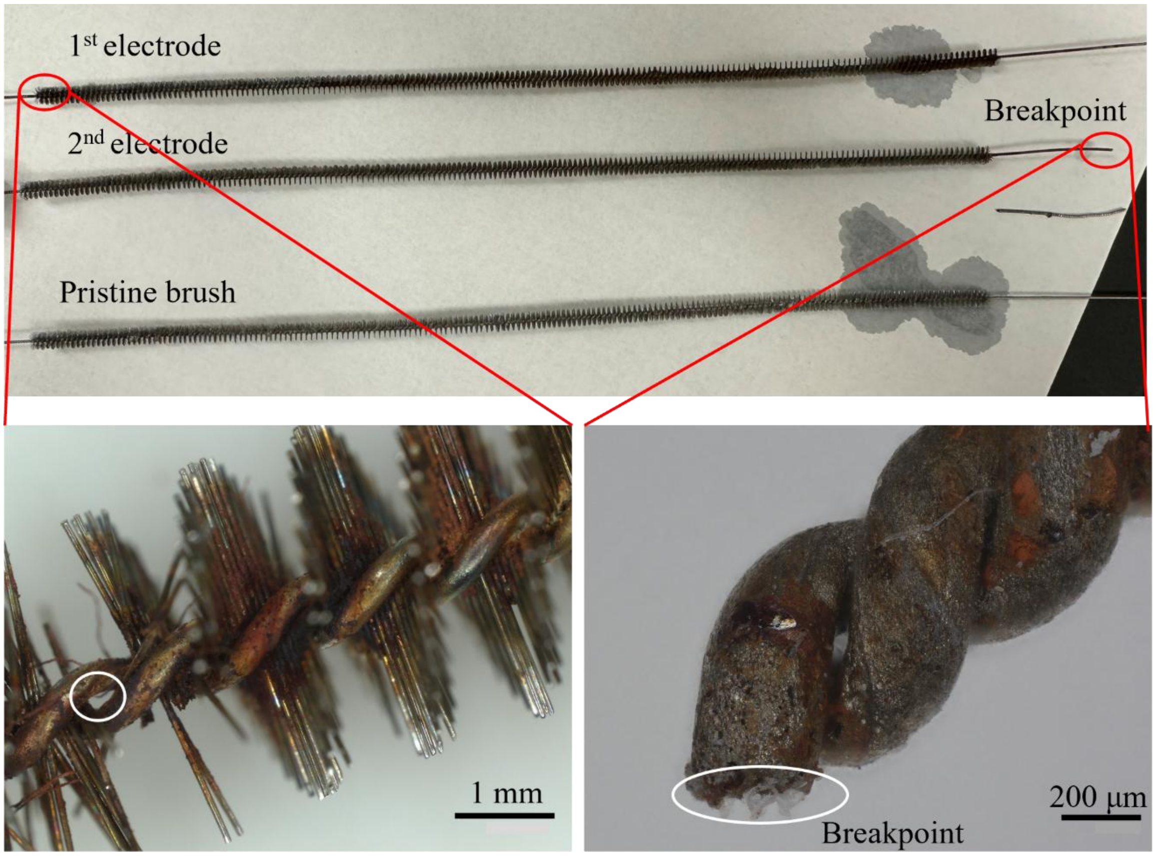

Figure S12. The images of the brush electrodes post LEEFT. The white circles show the loosen structure and breakpoint of the brush electrode.

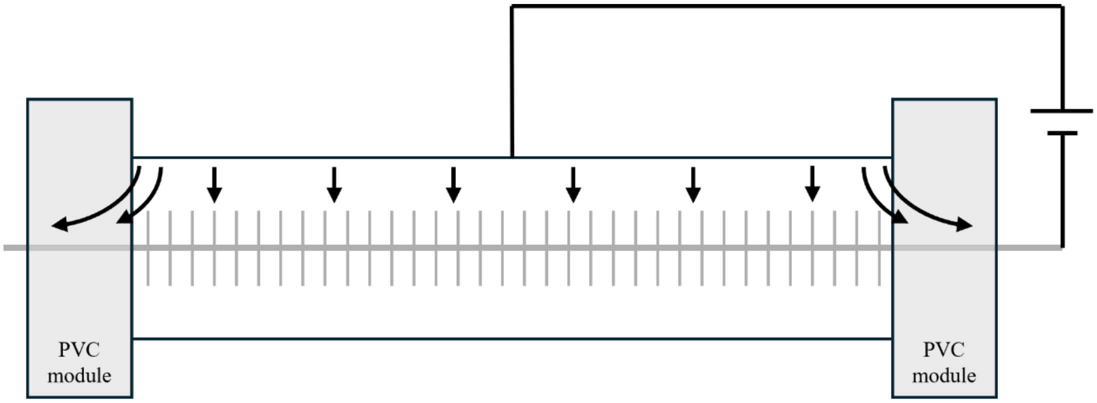

Figure S13. The schematic of the current flow (black arrows) within the tubular reactor.

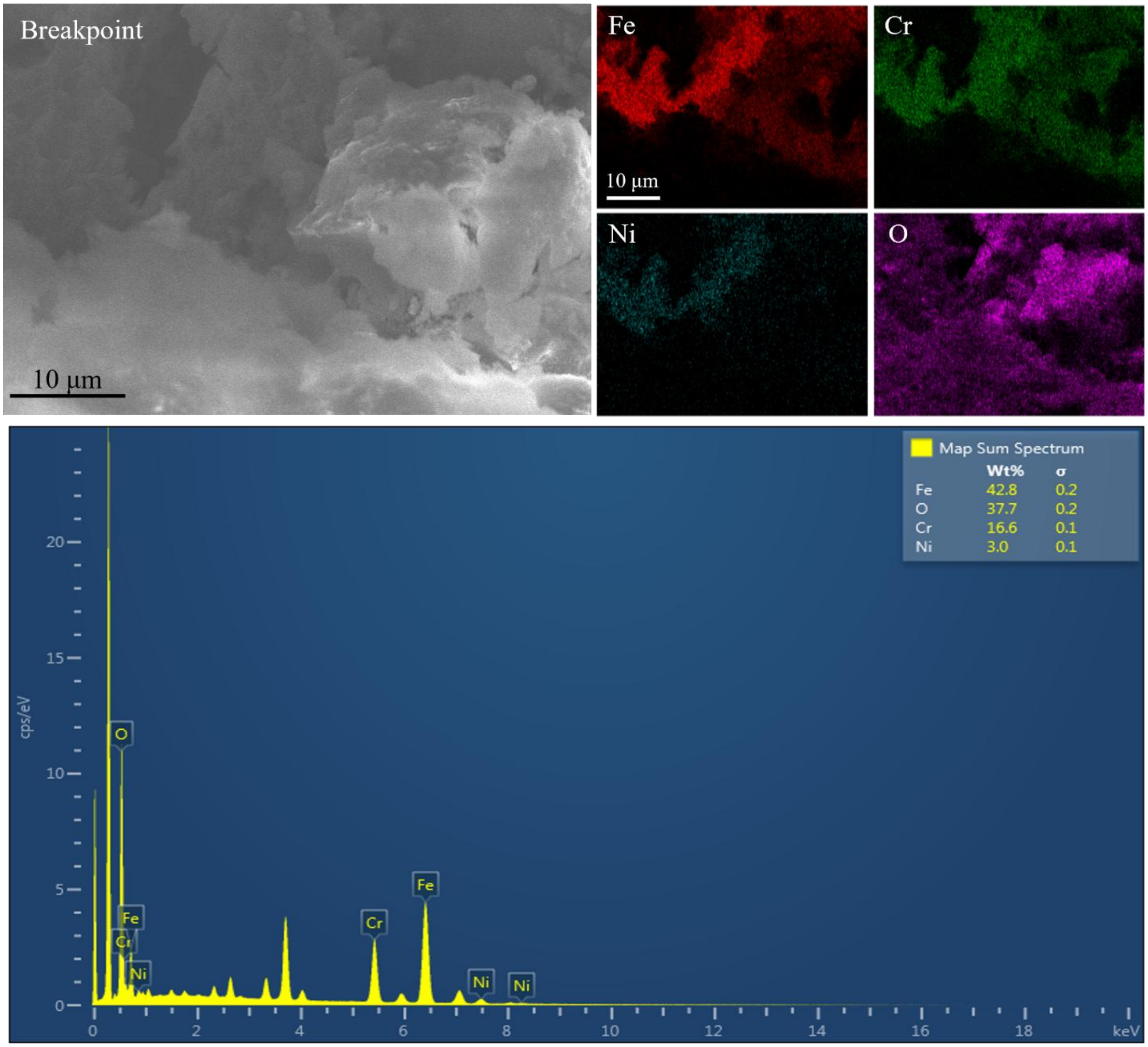

Figure S14. SEM-EDS analysis of the broken electrode at the breakpoint.

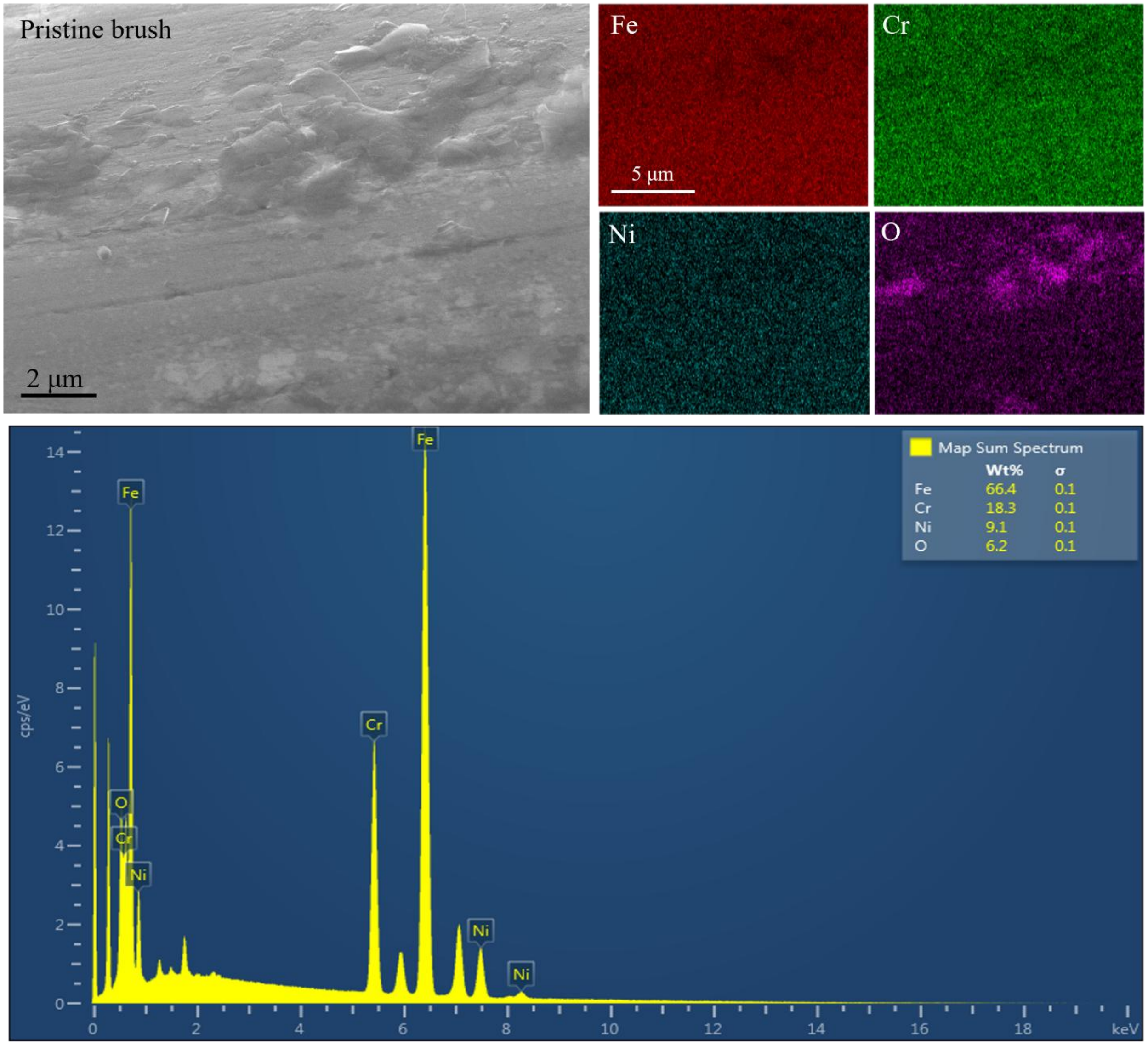

Figure S15. SEM-EDS analysis of the pristine electrode.

References

(1) Zhou, J.; Wang, T.; Xie, X. Locally Enhanced Electric Field Treatment (LEEFT) Promotes the Performance of Ozonation for Bacteria Inactivation by Disrupting the Cell Membrane. *Environmental Science & Technology* **2020**, *54* (21), 14017-14025.

(2) Huo, Z.-Y.; Xie, X.; Yu, T.; Lu, Y.; Feng, C.; Hu, H.-Y. Nanowire-Modified Three-Dimensional Electrode Enabling Low-Voltage Electroporation for Water Disinfection. *Environmental Science & Technology* **2016**, *50* (14), 7641-7649.

(3) Zhou, J.; Wang, T.; Xie, X. Rationally designed tubular coaxial-electrode copper ionization cells (CECICs) harnessing non-uniform electric field for efficient water disinfection. *Environment International* **2019**, *128*, 30-36.
